# Posterior Sternoclavicular Joint Dislocation in a Pediatric Wrestler: Delayed Diagnosis After Initially Benign Radiographs

**DOI:** 10.7759/cureus.109887

**Published:** 2026-05-29

**Authors:** Andrew Lew, Chace Davies, Suleman Janjua, Wardha Shabbir, Tariq M Awan, Royal Cole, Nicholas Moore

**Affiliations:** 1 Family Medicine, University of Nevada, Reno, USA; 2 Family Medicine, McLaren Oakland Family Medicine Residency Program, Pontiac, USA; 3 Sports Medicine, STEM Health, Frankenmuth, USA; 4 Sports Medicine, Ascension Michigan, Warren, USA; 5 Sports Medicine, Ascension Providence Hospital, Southfield, USA; 6 Family Medicine, Reno School of Medicine, University of Nevada, Reno, USA

**Keywords:** high energy trauma, open reduction, posterior sternoclavicular joint dislocation, sternoclavicular joint dislocation, wrestling

## Abstract

Posterior sternoclavicular joint (SC) dislocation is a rare but potentially life-threatening injury in pediatric athletes due to its proximity to mediastinal structures and frequent delay in diagnosis. We present a previously healthy 13-year-old male wrestler who developed persistent right SC pain and swelling after a forceful shoulder impact during a match, initially diagnosed as an acromioclavicular (AC) joint injury based on plain radiographs. Despite conservative management, continued symptoms prompted further assessment with computed tomography, which revealed a posterior SC joint dislocation. The patient underwent successful open reduction and stabilization without complications and progressed appropriately postoperatively. This case highlights the importance of maintaining a high index of suspicion and obtaining timely advanced imaging in young athletes with persistent SC pain following high-energy trauma to prevent serious complications and ensure safe return to sport. Though not seen in this patient, posterior SC dislocation can lead to cardiovascular, neurovascular, or airway compromise. Despite the initially reassuring shoulder X-ray, the mechanism of injury and severity of intractable pain ultimately led to the final diagnosis.

## Introduction

Posterior sternoclavicular (SC) joint dislocation is a rare injury, accounting for less than 1% of clavicular injuries, but carries significant morbidity due to its proximity to critical mediastinal structures [1,2]. The SC joint lies adjacent to major intrathoracic vasculature, including the brachiocephalic (innominate) vein, as well as the trachea and esophagus, making posterior displacement potentially life-threatening [1].

This injury most commonly results from high-energy trauma, such as motor vehicle collisions or contact sports. The typical mechanism involves a compressive lateral force to the shoulder that drives the clavicle medially and posteriorly behind the manubrium, although it may also occur from a direct anterior blow to the medial clavicle. While usually associated with high-energy mechanisms, posterior SC dislocations have also been reported after lower-energy falls or sports-related impacts, necessitating a high level of suspicion from evaluating clinicians [3,4].

Patients may present with localized SC pain, swelling, and decreased shoulder range of motion. Concerning symptoms include dyspnea, dysphagia, ipsilateral upper extremity swelling or cyanosis, paresthesias, weakness, venous congestion, diminished pulses, or worsening pain in the supine position, all of which may indicate mediastinal compromise [5]. On physical examination, tenderness over the SC joint is common, and posterior displacement may be suggested by decreased medial clavicular prominence, prominence changes with arm abduction or elevation, neurologic deficits, or vascular findings [6].

Plain radiographs, including serendipity (Rockwood) view, are often inconclusive due to overlapping mediastinal structures. Computed tomography (CT) is the imaging modality of choice, as it provides detailed evaluation of joint alignment, degree of displacement, and adjacent soft tissues. When vascular injury is suspected, CT angiography is recommended to assess the brachiocephalic vessels and exclude active bleeding or compression [1,2,7].

## Case presentation

We present a previously healthy 13-year-old male middle school wrestler who developed right shoulder pain after a match in which his opponent maneuvered and forcefully slammed his shoulder onto the mat. He was presented to the Ascension Providence Hospital's emergency department immediately after the injury. Plain radiographs were interpreted as showing an acromioclavicular (AC) joint injury; no other imaging was performed. He was discharged with conservative management, including sling immobilization, acetaminophen, and ibuprofen.

Five days later, he presented to the sports medicine clinic with persistent 5/10 sharp pain localized to the SC joint, radiating to the neck and posterior shoulder. He also reported swelling over the SC joint. Despite daily sling use and anti-inflammatory medications, he experienced minimal relief. His symptoms were exacerbated by movement and improved with rest.

On physical examination, there was visible swelling and tenderness to palpation over the SC joint. Active range of motion demonstrated 180 degrees of forward flexion, 160 degrees of abduction, 70 degrees of external rotation, and internal rotation to T6. The cross-arm test and Allen's test were positive. Stability testing, including load and shift, sulcus sign, apprehension, and relocation tests, was negative. Impingement testing (Hawkins and Neer) and labral and biceps testing (O’Brien, crank, Speed, and Yergason tests) were negative. Radial pulses, strength, and sensation were intact bilaterally, without gross deformity or muscle atrophy.

Repeat anteroposterior radiographs of the AC joint did not reveal acute abnormalities (Figure 1). However, given persistent pain and clinical concern, a CT of the thorax was obtained, which demonstrated a posterior dislocation of the SC joint (Figures 2, 3).

**Figure 1:**
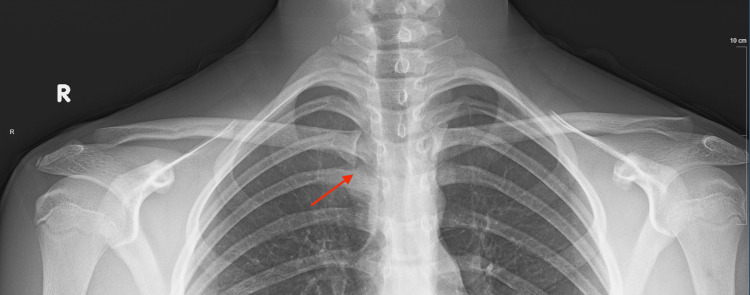
Preoperative chest radiograph demonstrating an asymmetrically widened right sternoclavicular joint (arrow).

**Figure 2:**
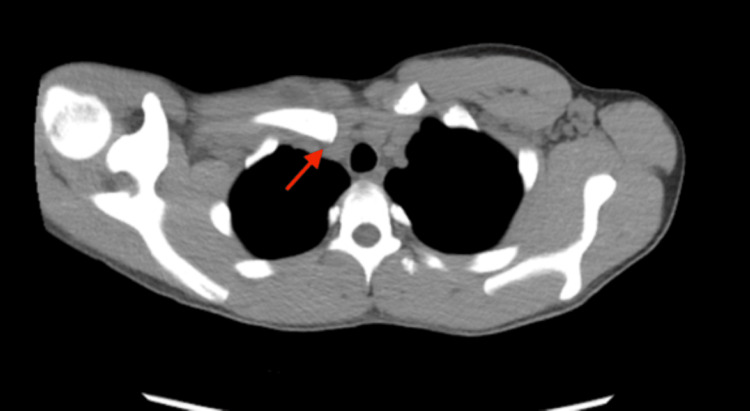
Preoperative axial CT demonstrating posterior displacement of the right clavicle (arrow), confirming posterior sternoclavicular joint dislocation.

**Figure 3:**
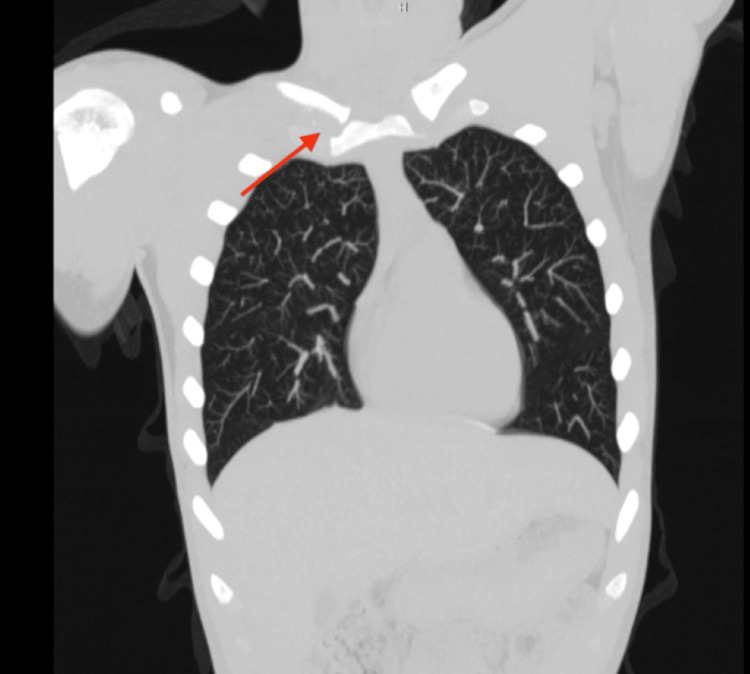
Preoperative coronal CT imaging demonstrating posterior displacement/dislocation of the right clavicle (arrow).

The patient underwent open reduction the following day, with the clavicle sutured to the sternum for stabilization. Postoperatively, he was treated with antibiotics, sling immobilization, strict non-weight-bearing precautions for the affected upper extremity, and a pain management regimen. He was instructed to avoid overhead shoulder movements for three weeks and began home exercises focused on elbow range of motion. At follow-up, repeat bilateral SC joint radiographs demonstrated appropriate alignment and a well-appearing reduction (Figure 4). At his two-month follow-up, he has minimal pain with a near full range of motion.

**Figure 4:**
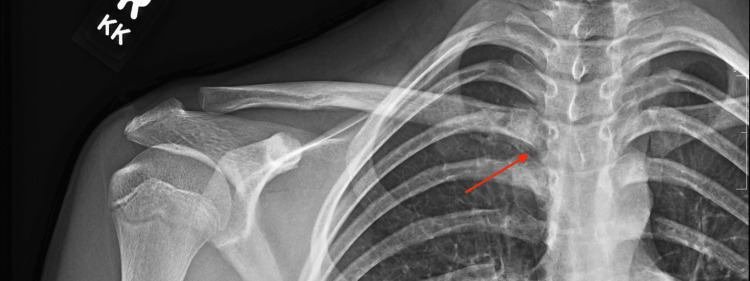
Postoperative radiograph demonstrating reduction of the sternoclavicular joint, now symmetric with the contralateral joint space (arrow).

## Discussion

Posterior SC joint dislocation is a rare injury with potentially serious complications, accounting for less than 1% of clavicle injuries in children. It usually results from high-energy trauma, such as contact sports, with lateral compression or direct shoulder impact [3,4]. Given the rarity of a posterior SC joint dislocation, it may not initially be suspected, and the diagnosis is often delayed.

While rare, a posterior SC joint dislocation is often considered a medical emergency that can compress the trachea, esophagus, or great vessels. Prompt recognition is vital due to the risk to mediastinal structures. Physical exam, including vitals, is often considered efficient to assess for airway or mediastinal compromise. Posterior SC dislocations are significantly more dangerous than anterior ones because the medial end of the clavicle is pushed backward into the superior mediastinum, an area packed with absolutely necessary vital structures [5]. Airway compromise generally occurs from a displaced SC bone, which can lead to dyspnea, stridor, or hoarseness. Esophageal compromise is due to a similar mechanism and can present with dysphagia, globus sensation, or odynophagia. The incidence of these complications is not known in the current literature. Injury to mediastinal structures is more common in instances of delayed diagnosis, further highlighting the need for prompt recognition and management. Rarely, they can cause life-threatening complications such as a tracheoesophageal fistula. Vascular injuries can arise from compression or laceration of the surrounding vessels. This may lead to significant bleeding, reduced pulses, and even shock. Additionally, the Allen’s test can be utilized, in which a positive test indicates good collateral flow (flush returns in <7-10 seconds). Thorough visual inspection is critical in the initial evaluation. SC joint asymmetry, palpable depression, or loss of normal medial clavicular prominence should prompt further evaluation. Crepitus in the area of the sternum may also be seen, as pneumomediastinum [8].

Further, an evaluation for dermatomal sensation can aid in the identification of SC joint dislocations, as it can help identify neurological deficits caused by compression of the brachial plexus or nerve roots. Key areas to test include the C5 (clavicle/shoulder), C6 (lateral arm), and T1-T2 (medial arm/axilla) dermatomes using light touch and pinprick, comparing the injured side to the contralateral side. The clavicle, shoulder, and lateral arm should be evaluated, especially if there is suspicion for a superior SC dislocation, which may cause traction on the brachial plexus. The medial arm and axilla may indicate if a posterior SC dislocation causes compression behind the clavicle. Testing can be done by initially assessing for light touch and pain (pinprick) sensations, with bilateral dermatomal comparison to identify numbness or hyperesthesia.

In addition to clinical symptoms and physical examination, imaging may help with making the diagnosis, with CT as the imaging of choice, and MRI to help with distinguishing physeal involvement. In terms of treatment, closed reduction within 48 hours is preferred, with open reduction reserved for unstable or failed cases. Outcomes are excellent, with most patients returning to sport in about three months, though no standardized return-to-play protocol exists [7]. An expected timeline for resolution of symptoms is not established due to the paucity of evidence.

Management depends on timing and associated complications. For uncomplicated atraumatic subluxations, nonoperative management with a shoulder sling can be an appropriate initial option [9,10]. One study demonstrated 25% patients with uncomplicated traumatic subluxations who underwent nonoperative management later required procedural intervention, most frequently as a result of joint instability [9]. Otherwise, closed reduction is recommended, with reported success rates ranging from approximately 38%-50% in adult studies [1,5]. Some literature supports attempts at reduction up to 7-10 days, and occasionally up to three weeks, though delayed cases are more complex due to callus formation and potential adherence to mediastinal structures [1]. This is performed with the abduction traction technique, where the supine patient is positioned with their dislocated shoulder near the edge of the table, with a 3- to 4-inch thick sandbag placed between their shoulders. Lateral traction is applied to the abducted arm and encouraged into extension. Imaging is repeated with an intraoperative X-ray aimed 40 degrees cephalad at the manubrium. Postoperatively, this will be confirmed with CT. Research varies, though this technique has been performed without a thoracic surgeon present [1,5].

Open reduction is indicated for failed closed reduction, mediastinal compression, or vascular injury, and late presentations over 10 days, where callus formation could cause adherence to underlying vessels. Reconstruction techniques include biologic autografts (palmaris longus, plantaris, or hamstring), allograft reconstruction, or synthetic fixation methods [1]. Notably, posterior reduction carries bleeding risk due to potential loss of the tamponade effect on compressed great vessels, and some authors recommend cardiothoracic surgical availability during reduction attempts [1,2]. Given the risk of life-threatening complications, prompt recognition and appropriate imaging are essential in any patient with persistent SC pain following shoulder trauma.

We present a case in which a patient was initially not suspected to have sustained a serious injury but was subsequently diagnosed with a posterior SC joint dislocation requiring operative management. In this case, persistent pain prompted further evaluation and advanced imaging with a CT, ultimately leading to definitive treatment. Further evaluation with CT angiography could have been considered as well; however, given the lack of cardiovascular involvement and the prompt surgical procedure, it was determined to be not required. Fortunately, the patient recovered well without complications and returned to sports successfully. However, the details of the exact mechanism of the impact during the wrestling match are limited due to potential recall bias.

Due to the extreme rarity of posterior SC joint dislocations, the management strategies described here are based on a single instance rather than large-scale, comparative evidence. The scarcity of literature makes establishing a standardized consensus on treatment difficult, highlighting the need for caution in applying this approach to all patients. There are no dedicated society-published clinical practice guidelines for the evaluation and management of this injury. The American Academy of Family Physicians (AAFP) briefly addresses posterior SC joint dislocation in its 2023 guideline on acute shoulder injuries but only goes so far as to designate it as a medical emergency requiring CT evaluation and prompt surgical intervention [11].

## Conclusions

Posterior SC joint dislocations represent a potentially life-threatening injury due to their proximity to vital mediastinal structures. This case highlights the importance of maintaining a high index of suspicion in young athletes presenting with persistent SC pain following high-energy shoulder trauma, even when initial radiographs are interpreted as benign. Normal or inconclusive plain films do not exclude significant SC pathology.

Early recognition, appropriate advanced imaging with CT, and timely surgical intervention are critical to preventing complications and optimizing outcomes. Persistent pain, focal SC swelling, and the mechanism of injury should prompt further evaluation. With prompt diagnosis and management, patients can achieve excellent functional recovery and a successful return to sport.
